# NEIGHBORHOOD ENVIRONMENT AND LONELINESS IN LATER LIFE: DO PERCEIVED EXPERIENCES MEDIATE THE OBJECTIVE FEATURES?

**DOI:** 10.1093/geroni/igae098.1533

**Published:** 2024-12-31

**Authors:** Reza Tayari Ashtiani, Sara Moorman

**Affiliations:** Boston College, Chestnut Hill, Massachusetts, United States; Boston College, Chestnut Hill, Massachusetts, United States

## Abstract

The health of older adults may be more impacted by their neighborhoods due to limited mobility. Thus, the attributes and quality of the neighborhood environment are crucial for the social life and health outcomes of older individuals. This study aims to examine the role of neighborhood factors in loneliness, investigating associations between objective residential environment, perceived neighborhood cohesion and danger, and loneliness. We used cross-sectional data from 4,377 midlife and older adults (49–95 years) participating in the National Social Life, Health, and Aging Project (NSHAP). We measured the residential environment using a scale based on the interviewer’s observation of the neighborhood’s quality. The scale included five items: the street’s cleanliness, noise, amount of traffic, closeness of houses together, and strong smell or air pollution. We employed multiple regression models. The product of coefficients tests showed that while neighborhood residential environment was not directly associated with loneliness, it was significantly associated indirectly, through both perceived social cohesion (B = 0.006, p <.05) and perceived neighborhood danger (B = 0.010, p <.05). In turn, perceived neighborhood social cohesion was negatively associated with loneliness (B = -0.13, p <.001), whereas perceived neighborhood danger was positively associated with loneliness (B = 0.05, p <.05). These findings underscore the significance of incorporating both objective and subjective elements of neighborhood environments in relation to the experience of loneliness.

